# Light People: Professor Stefan Hell

**DOI:** 10.1038/s41377-022-01034-w

**Published:** 2022-11-22

**Authors:** Hui Wang

**Affiliations:** Changchun Institute of Optics, Fine Mechanics and Physics, Chinese Academy of Sciences, 3888 Dong Nan Hu Road, 130033 Changchun, China

**Keywords:** Optical spectroscopy, Imaging and sensing

## Abstract

“When all those around me are drunk, I alone am sober,” lamented an ancient Chinese poet on fighting a lone and helpless cause. In the world of science a few decades ago, there was also a lone researcher who pursued the field of optical resolution despite suspicions and derisions. Unlike those who made their names young, this scientist only succeeded thanks to his own perseverance. He developed the 4Pi microscope and successfully increased the vertical resolution of traditional optical microscopes by 3–7 times. Once he sold the patent to a company, he invested the little personal money gained from the patent in breaking the Abbe diffraction limit. Despite all his hard work, his papers kept getting rejected by respected journals, and his work was subjected to mistrust and even criticism. This scientist is Stefan Hell, someone who is never afraid of difficulties and ever determined to forge ahead.

Hell was awarded the Nobel Prize in Chemistry in 2014 for being the first to propose and demonstrate that the optical diffraction limit can be broken, and for successfully developing the STED super-resolution fluorescence microscope. Later Hell and his colleagues proposed and commercialized MINFLUX, which brought optical microscopy technology to the three-dimensional single-nanometer scale, opening the “post-superresolution era”.

Frank and sincere, persistent and humble, boldly innovative and resolute, Hell is a scientist, an entrepreneur, a mentor, and an everyday man who enjoys life.

In this interview, we will reacquaint ourselves with the Nobel laureate, Stefan Hell.

## Abstract (web summary)

Prof. Stefan Hell was awarded the Nobel Prize in Chemistry in 2014 for being the first to propose that the optical diffraction limit can be broken, and for successfully developing the STED super-resolution fluorescence microscope.

## Graphical abstract

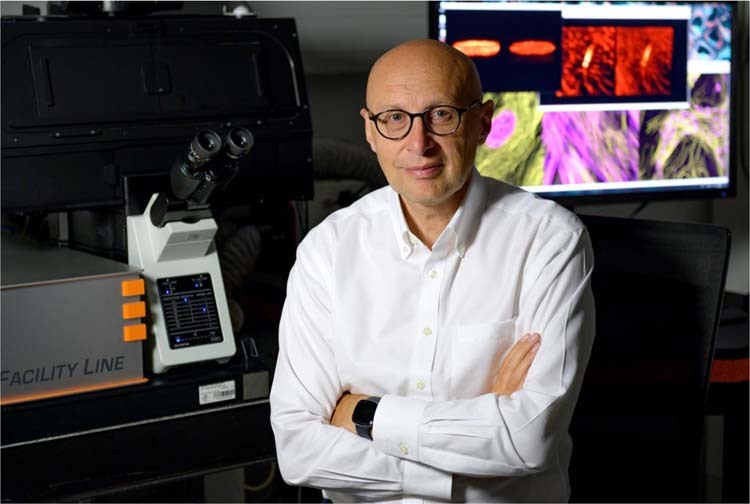

**Biography:** Stefan Hell is a director at both the Max Planck Institute for Multidisciplinary Sciences in Göttingen and the Max Planck Institute for Medical Research in Heidelberg, Germany.

**Figure:**
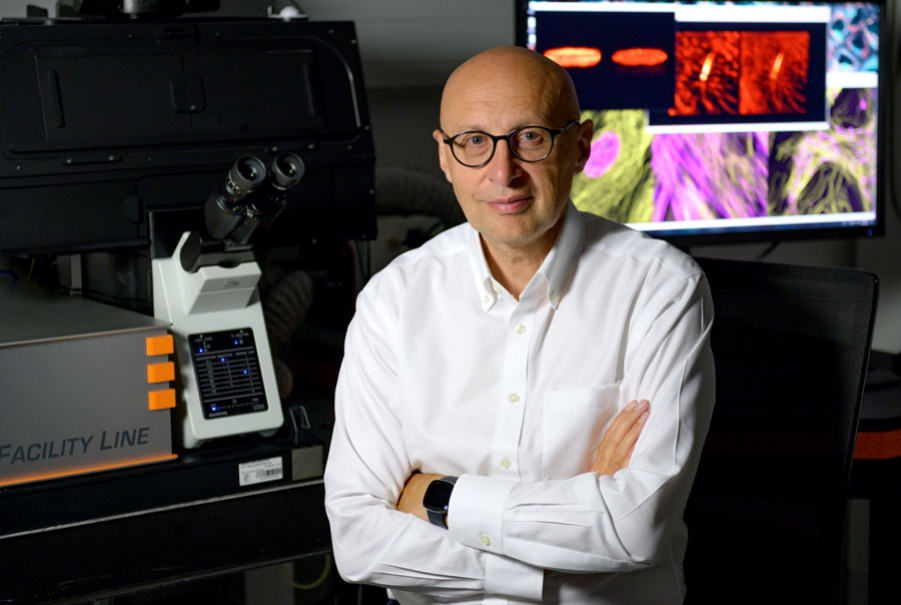

He is credited with having conceived, validated, and applied the first viable concept for overcoming Abbe’s diffraction-limited resolution barrier in a light-focusing fluorescence microscope. For this accomplishment, he has received numerous awards, including the 2014 Kavli Prize in Nanoscience and the Nobel Prize in Chemistry.

Stefan Hell received his doctorate (1990) in physics from the University of Heidelberg. From 1991 to 1993 he worked at the European Molecular Biology Laboratory, followed by stays as a senior researcher at the University of Turku, Finland, between 1993 and 1996, and as a visiting scientist at the University of Oxford, England, in 1994. In 1997 he was appointed to the MPI for Biophysical Chemistry (named Max Planck Institute for Multidisciplinary Sciences since 2022) in Göttingen as a group leader and was promoted to director in 2002. From 2003 to 2017 he also led a research group at the German Cancer Research Center. Hell holds honorary professorships in physics at the Universities of Heidelberg and Göttingen.

**1. You first proposed MINFLUX, a super-resolution display technology based on minimum radiation flux, which marks the start of the “post-super-resolution era”. MINFLUX successfully brought optical microscopy technology to the three-dimensional single digital nanometer scale. Could you briefly introduce this technology and its future development trend?**

Prof. Hell: MINFLUX is a real game changer in two very important fields: superresolution fluorescence microscopy (nanoscopy) and single fluorophore tracking. I firmly believe that MINFLUX is a seminal concept that will go down in the history of light microscopy. In that sense, you are right that it can be perceived as the start of a new era. The reason why MINFLUX fluorescence nanoscopy is so powerful is that it synergistically combines the strong points of STED and PALM/STORM in order to attain the highest resolution possible in fluorescence microscopy: resolution at the molecular (1–3 nm) scale. MINFLUX separates the fluorophores on a single molecule basis like PALM, but uses a light field having a central intensity minimum such as a donut, like in STED, to localize the emitting fluorophore faster and/or with higher precision. For localizing with a precision of 1 nm, MINFLUX needs 100–300 times fewer fluorescence photons than a typical PALM/STORM arrangement. This is why MINFLUX will also change the field of single-molecule localization in biophysics and single-molecule biochemistry. A first impressive example has just been given by the recent study of how kinesin-1 walks on microtubules. Please, see 10.1101/2022.07.25.501426v1.

There is definitely more in the pipeline… Stay tuned!

**2. You are the first person in the world to propose breaking the diffraction limit to achieve super-resolution. What inspired you?**

Prof. Hell: After I had finished my Ph.D. studies at the University of Heidelberg, I had the wish to do something that is truly unique and unexpected in science. I wanted to accomplish something that would make the world listen. I didn’t want to be a scientist just for the purpose of being a scientist. I wanted to make a difference as a person. I also felt that doing it in an area of science that was considered ‘closed’ or not ‘timely’—in fact, this was the situation of light microscopy at the end of the 20^th^ century—would give me an even greater opportunity to do something profound. If you work in a rather crowded field of science, then the chances of making a difference as an individual are not great. It’s just like digging for gold where many other people dig as well. It’s better to look around and find a place where no one else digs but where one still has a reasonable chance of finding a nugget in the end. Of course, it is not always easy to find that area and, besides skill and intuition, this strategy also needs a bit of luck.

**3. When you were looking for ways to break the optical diffraction limit, I think it’s fair to say that much doubt was casted your way. Some people even went so far as to say that your work was not meaningful for improving the resolution of optical microscopes. How did you deal with those criticisms? Looking back now, what did that period mean to you?**

Prof. Hell: This is correct. In fact, some people went so far as to state both at meetings and in papers that I exaggerate or that my claims were false. These people got more silent over time but none of them later frankly admitted that they were wrong, let alone apologize. This is an interesting psychological phenomenon. People with strong negative opinions hardly change their views. Scientific progress does not evolve by people admitting that they were wrong, but by the next generation of scientists taking up new ideas very naturally. The opponents of a new discovery or development simply disappear over time. By the way, this was already noted by Max Planck, the German Nobel prize winner, who laid the first cornerstone of quantum mechanics. When I started the fluorescence nanoscopy field in about 1989–1994, I did not know that I would eventually win a Nobel prize. Actually, if you had had the opportunity to ask me at that time about whether I would think that I would become a famous scientist, I would have laughed. Back then, I was unable to get my papers published in a ‘reputable’ scientific journal. However, I carried on because I felt that I had reasonable scientific arguments and I really enjoyed my research. I also believe in human ingenuity. One should never underestimate how creative humans are when they are determined to solve a specific problem.

**4. In 2014, you were awarded the Nobel Prize in Chemistry for your successful development of a super-resolution fluorescence microscope. What does this award mean to you? Has it changed you in anyway?**

Prof. Hell: It has been a huge recognition for me and all the people that have worked in my laboratory. At the same time, the Nobel Prize was a major encouragement to go a big step further and develop MINFLUX. It was my intention to show that superresolution had not reached its limits in 2014, but had just taken off. With my research group, I wanted to be the first to get down to the 1–2 nm resolution scale, i.e. the size of the fluorophore. Now it is here.

**Figure:**
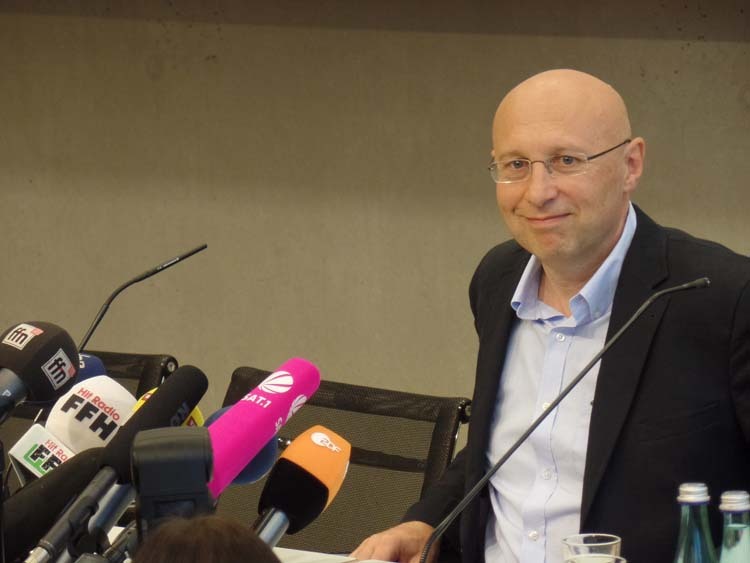
Prof. Hell answering questions from journalists on 08 October 2014 on Nobel Prize

**Figure:**
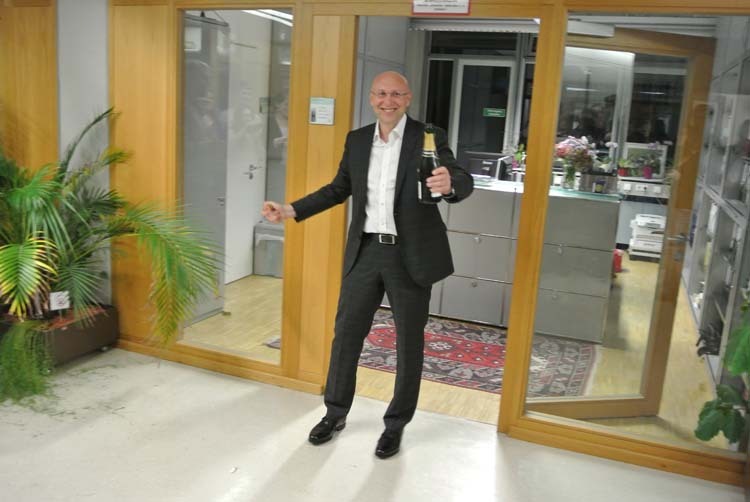
Prof. Hell opening Champagne to celebrate his Nobel Prize

**Figure:**
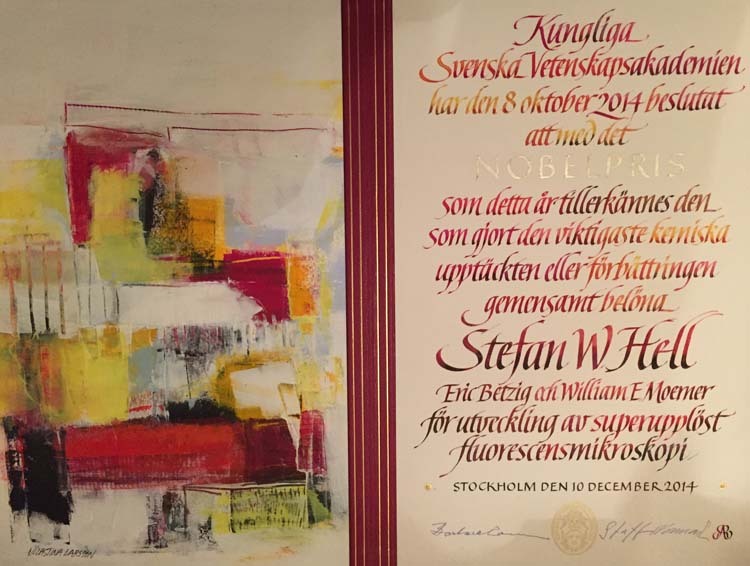
Prof. Hell’s Nobel Award Certificate

**5. Not long ago, you gave an excellent talk on iCANX at the invitation of Professor Peng Xi of Peking University, and it was watched by at least 37,800 people with a huge response. Prof. Peng Xi worked in your lab as a visiting scholar and continued to work on STED after returning to China. How do you see China’s development in this field?**

Prof. Hell: I have no doubt that China will become a leading nation in science in the 21^st^ century. China has all the prerequisites for reaching this status, namely many talented and highly motivated young scientists who want to make a difference in their lives. It reminds me of Germany in the late 19^th^ and early 20^th^ century when the country was on an upward economic and political trajectory, rivaling the United Kingdom and France. On the other hand, the example of Germany shows that the newly assumed leadership has to be used wisely. I am confident that China can live up to this important role, because unlike Germany, which was a very young nation at that time, China is the nation with the longest continual history in the world.

**6. Today, the STED microscope has been successfully commercialized, and Abberior Instruments has been established, specializing in microscope systems. Its achievements have been applied in the fields of biomolecules and neuroscience, bringing technological innovations to mankind. In addition, you have also established the Abberior-Labels company. How are the businesses of your two companies connected? What do you think should be the relationship between scientific research results and their commercialization?**

Prof. Hell: I am convinced that all fundamental scientific discoveries have commercial implications. In some cases, it may take time until the commercial relevance becomes apparent, whereas in other cases this is instantly obvious. However, one should not underestimate the very positive reciprocal impact that commercialization has on science. After all, commercialization creates tax revenue and this is used to drive science in turn. If you look back in history, strong national economies have always gone hand in hand with strong global positions in science. The reason why I have set up a label and fluorophore company is that the fluorophores and their features are crucial for the resolution and contrast that is produced by a superresolution microscope. If you ask me, my actual scientific discovery was that the energy states of fluorophores lend themselves to overcoming the diffraction barrier. I realized that the fluorophore states—not the modification of the propagation of light waves—are the key to breaking the diffraction barrier. At the time I conceived STED microscopy—which is purely based on separating fluorophores by preparing them in different states- other scientists, most prominently Eric Betzig—developed near-field optical microscopy, which relied on squeezing the light waves through nanosized openings. I knew back then that this was an inefficient concept. Hence, in my quest for the highest possible resolution, fluorophores have always been on center stage. Consequently, I founded a fluorophore-making company that aims at providing the best possible fluorophores for the best possible superresolution fluorescence imaging.

**7. You once said that “every confocal system should be a STED system.” From a historical perspective, could you explain the relationship between STED technology and confocal systems?**

Prof. Hell: As a Ph.D. student in the late 1980s, I worked with confocal microscopy which was relatively new and modern light microscopy at that time. I also realized that overcoming the diffraction barrier will be technically easier in a single-point scanning system compared to a widefield or multipoint scanning microscope, because of the simple fact that such a scanning microscope images only one point at a time. I thought that once the problem is solved for single point imaging, one can then apply the principle ‘in parallel’ to many points at the same time. In addition, confocal pinholes provide efficient suppression of background which is always a problem when you look for new optical effects and develop a new measurement device. That’s why I started out with the confocal microscope. It’s not that I thought that in a widefield microscope it would not work. I just thought it would be easier in confocal. Still, to this very day, I believe that standard STED microscopy owes its unique ‘push-button-and-get-a-superresolution-image’ functionality to the fact that it is implemented in a confocal scanning system. And yes, I still believe that every single laser-beam-scanning confocal system must have a ‘STED button’ to be up-to-date and versatile.

**8. You have been working on STED technology since 1994. What is the attraction? What does optics mean to you?**

Prof. Hell: The attraction has been the simple but fundamental fact that I had found a way to overcome the diffraction barrier in a light-focusing microscope and that this would open a new chapter in light microscopy and a new field. Keep in mind that in 1994 other people who tried to overcome the diffraction barrier, including Eric Betzig, worked on so-called near-field optics, where the light is squeezed through a tiny tip of a pulled glass fiber. I knew already at that time that this was not the way to go. What I had discovered was that the key to overcoming the diffraction barrier was not the modification of the light propagation but that of the occupation of the fluorophore states. I knew that this was a major discovery and this is why I kept going.

**Figure:**
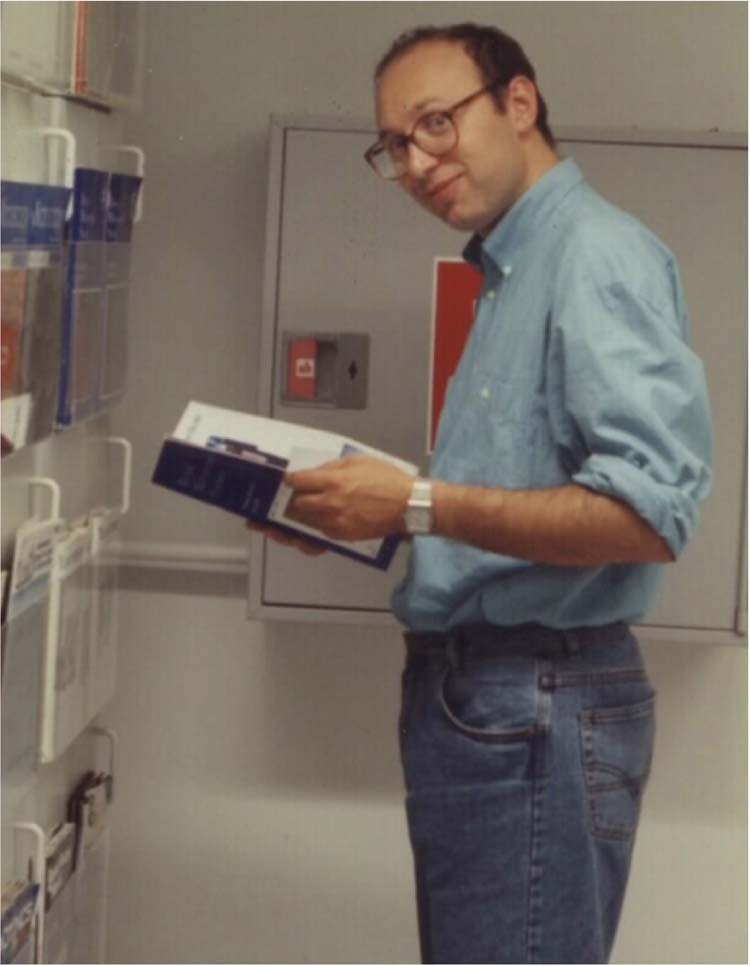
Prof. Hell in 1993 around the time of STED invention

**9. What career would you have gone for if you didn’t choose to do research?**

Prof. Hell: Hard to tell. Honestly, I don’t know. I have not been in the situation to think about it.

**10. Your career paths once covered the Heidelberg University in Germany, the University of Turku in Finland, and Oxford University in England, etc. What do you think are the similarities and differences in teaching and research in these countries? How have these different experiences affected you?**

Prof. Hell: Germany at that time, and probably until the very day, had a very liberal university system where one could take as much time as one needed to study a certain topic. Everything was free; there were no tuition fees. Moreover, we had no written exams, only oral ones. The downside of that was that many students, particularly those with low personal motivation, did not get educated well enough. However, those who wanted to make a difference were given enough freedom and time to set up their own view and interpretations of the various physics subjects. Thus good students could establish strong and highly original personal skills. I could succeed because I was inherently forced to set up my own, and arguably superior interpretation of the diffraction barrier. Having understood it better than the rest, I could smash the barrier in the end.

**11. You are a scientist, an executive of the Max Planck Institute, and an entrepreneur. What do you think should be the relationship between these three roles? How do you juggle these roles?**

Prof. Hell: I am strictly separating these roles. In the first place, I am a scientist who tries to create new and impactful knowledge. That is my first priority. Full stop. All the inventions that I make belong 100% to the Max Planck Institute. I do very little administration, since in the Max Planck Institutes we have good administrators who take care of that. Most of the executives of Abberior and Abberior Instruments are my former students. I help them do their job, by meeting them briefly once or twice a week. I also encourage them to take licenses from the Max Planck Institutes if they think that the inventions are useful. However, it is fully up to them to decide on commercialization and I stay fully out of commercial decisions and negotiations.

**12. As a tutor, you have trained many students. What abilities do you focus more on cultivating students in your teaching?**

Prof. Hell: As long as they work in my laboratory, they work on problems that I prefer and prioritize. However, I strongly encourage them to come up with their scientific vision and set up their own independent laboratory.

**Figure:**
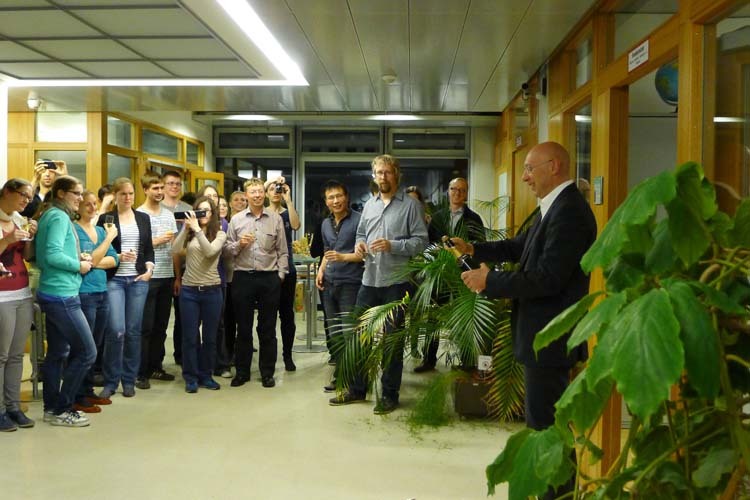
Prof. Hell opening Champagne with students to celebrate his Nobel Prize

**13. What was the most exciting moment in your research career?**

Prof. Hell: When I realized that the diffraction barrier will be smashed and that this will open the floodgates for eventually getting down to molecular scale resolution. Now, we are there with MINFLUX.

**14. You have visited China, so what similarities and differences do you see between the scientific research systems of China and Germany? What do you think of Chinese researchers? What do you think are their strong points and is anything needed to be improved? Do you have any plans to visit China again?**

Prof. Hell: I have great admiration for Chinese culture and Chinese researchers. I think their activities will transform the world in the 21^st^ century just as Western scientists did in the 20^th^ century. Nonetheless, the world of science is highly connected, and every part of the world will play its role. Surely, I will visit China since I love to talk to young scientists that will be the stars of the future.

**Figure:**
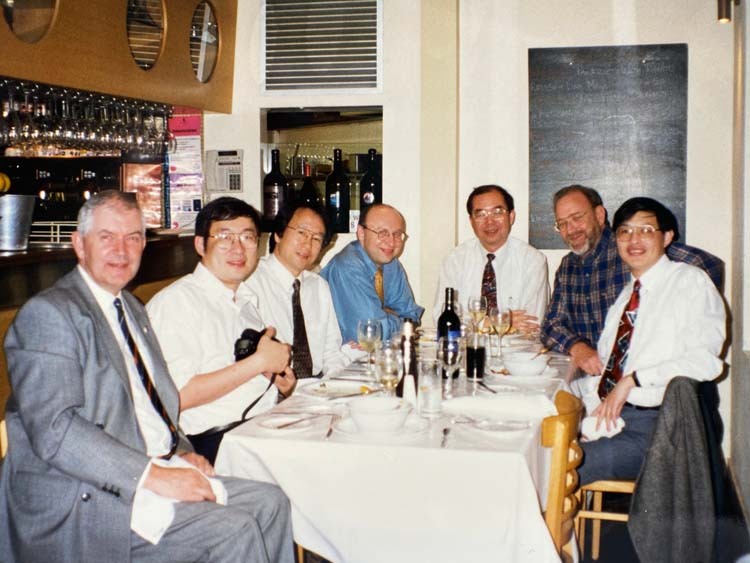
Prof. Hell (4^th^ right) at dinner with Prof. Min Gu (right, now USST Shanghai) and colleagues, April 1995

**Figure:**
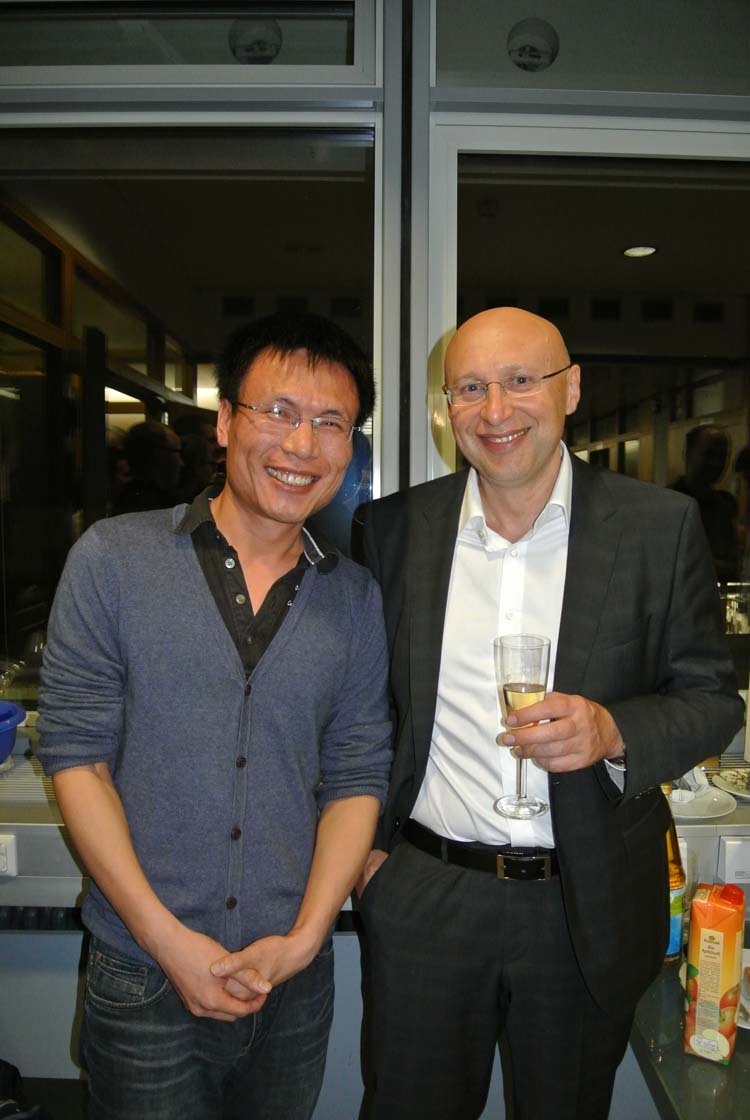
Prof. Hell with Chinese postdoc Haisen Ta at a party celebrating his Nobel win

**15. Has anyone had a major influence on you in your career? In what way?**

Prof. Hell: Often it is the adversaries that have a higher impact on one’s trajectory than the so-called mentors. Adversaries force you to prove them wrong.

**16. What’s the secret of your amazing creativity and innovativeness?**

Prof. Hell: I wish I knew. It is definitely not hard work. If work feels hard, please do something else.

**17. Many scientific researchers in China devote themselves to their work, often to the extent of sacrificing their own personal time or quality time with their family. How do you think scientists should balance professional and personal life?**

Prof. Hell: Again, do the work that you enjoy most and it does not feel hard. Only if you have the right balance between enjoyment and motivation you are creative.

**18. What are your hobbies?**

Prof. Hell: Playing football, i.e. soccer, with my boys.

**19. What advice and suggestions would you give our young audience on life and career?**

Prof. Hell: Aim high, stay grounded.

